# Innovations in geriatric care: leveraging technology to meet the needs of an aging world, a narrative review

**DOI:** 10.1186/s42506-025-00203-0

**Published:** 2026-02-06

**Authors:** Ayat Ashour, Ghada Othman Elkhawaga

**Affiliations:** 1 Family Health Department, High Institute of Public Health, Alexandria University, 165 El Horeya Avenue, Alexandria, 21561 Egypt; 2 Public Health, Family and Community Medicine Department, College of Medicine, Jouf University, Sakakah, Kingdom of Saudi Arabia

**Keywords:** Geriatric care, Digital health, Telemedicine, Wearable technologies, Artificial intelligence, mHealth, Aging population

## Abstract

The rapid global increase in the older adult population presents unprecedented challenges and opportunities for healthcare systems worldwide. This demographic shift is accompanied by a growing prevalence of chronic conditions such as cardiovascular diseases, diabetes, dementia, and other geriatric syndromes, underscoring the urgent need for innovative, age-sensitive healthcare solutions. Traditional healthcare models often fall short in addressing the complex, multifactorial needs of older adults, highlighting the need for a transformative approach to care delivery and management that integrates personalization, continuity, and technological innovation.

This article is a narrative review that synthesizes recent literature published within the last ten years, drawing from PubMed, Scopus, Web of Science, and Google Scholar to explore cutting-edge advancements in geriatric care, with a focus on telemedicine, mobile health (mHealth), artificial intelligence (AI), robotics, and wearable technologies. These innovations offer promising avenues to improve healthcare accessibility, enhance patient outcomes, and alleviate the burden on caregivers. For instance, telemedicine facilitates remote consultations, reducing hospital visits and ensuring continuity of care for individuals in remote or underserved areas. mHealth applications empower older adults to actively manage their health, monitor chronic conditions, and stay connected with caregivers. Robotics and AI-driven systems provide physical support, cognitive stimulation, and personalized care planning, thereby enhancing independence and emotional well-being. However, successfully integrating these technologies into geriatric care requires addressing several challenges, including technical limitations, data privacy concerns, and disparities in digital literacy. A major hurdle lies in ensuring that these solutions are user-friendly and tailored to the diverse physical and cognitive needs of older adults.

## Introduction

By 2070, the number of people aged 65 years and older globally is projected to reach 2.2 billion, surpassing the number of children under age 18, according to the United Nations World Population Prospects 2024 [1]. Age-related conditions such as dementia, diabetes, cardiovascular disease, and geriatric syndromes are becoming increasingly prevalent, creating a demand for integrated, adaptive, and person-centered care models [2]. Traditional healthcare systems, often constrained by workforce shortages and institutional limitations, are not fully equipped to meet the multifaceted needs of older adults. These challenges highlight the urgency of developing innovative solutions that extend care beyond conventional clinical settings and empower older adults and their caregivers. Technological advancements, including telemedicine, mobile health, artificial intelligence, and assistive devices, offer promising pathways to transform how geriatric care is delivered, monitored, and personalized [3].

## Materials & methods

This article is a narrative review that synthesizes recent evidence and illustrative examples of technological innovations in geriatric care. Although narrative reviews do not follow the strict protocol of systematic reviews, a transparent and structured search approach was adopted. A purposive search was conducted in PubMed, Scopus, Web of Science, and Google Scholar between October and December 2024, using combinations of keywords such as *“geriatric care*,*” “older adults*,*” “digital health*,*” “telemedicine*,*” “telehealth*,*” “mobile health (mHealth)*,*” “artificial intelligence AND older adults*,*” “machine learning*,*” “robotics AND elder care*,*” “assistive technology*,*” “wearable technology*,*” “remote patient monitoring*,*”* and *“smart home technologies”.* The search focused on literature published within the past ten years (2014–2024) and included peer-reviewed journal articles, authoritative organizational reports (e.g., WHO, UN, ITU), and relevant policy documents. Studies were included if they: (1) examined technological or digital innovations applied to older adults or geriatric care, (2) provided empirical evidence, conceptual insights, or practical applications, and (3) were written in English. Editorials, abstracts without full texts, and articles not focused on older adults were excluded.

Given the narrative nature of the review, the findings were synthesized thematically. The selected literature was organized around key thematic areas: telehealth, mobile health (mHealth), artificial intelligence (AI), robotics, and assistive technologies, to illustrate major trends, representative innovations, and emerging challenges. This approach provides an integrative overview of the evolving landscape of technological solutions in geriatric care rather than a systematic or quantitative evidence synthesis.

## Telehealth and remote health care

Telehealth, as defined by the World Health Organization (WHO), involves the use of information and communication technologies (ICTs) to provide remote healthcare services, encompassing diagnosis, treatment, prevention, education, and research. It supports universal health coverage by improving access to cost-effective, quality healthcare for people in remote or underserved areas, including vulnerable populations and older adults [4].

Although often used interchangeably, telehealth is a broader term than telemedicine. Telehealth includes clinical (e.g., consultations) and non-clinical services (e.g., education, administration). Telemedicine, a subset, focuses on delivering clinical care remotely, using technologies for real-time (synchronous) or delayed (asynchronous) communication [5].

Historically, telemedicine dates back to the 1920 s with radio-based consultations for ships. In the 1960 s, the Nebraska Psychiatric Institute launched a telepsychiatry program, and NASA developed remote care technologies for astronauts, laying the groundwork for modern telehealth. The internet revolutionized the field by enabling real-time video consultations and remote monitoring. More recently, mobile health (mHealth), artificial intelligence (AI), machine learning (ML), and the Internet of Things (IoT) have further advanced the reach and functionality of telemedicine platforms [6].

Teleconsultation involves real-time video or audio interactions between patients and providers, improving access to care without intermediaries. Remote patient monitoring tools (e.g., wearables) are often integrated into teleconsultation platforms to continuously track vital signs like heart rate and glucose levels [7]. A meta-analysis of 27 randomized trials found that synchronous teleconsultation for adults with type 2 diabetes significantly improved HbA1c but showed no meaningful effects on BMI, blood pressure, LDL-cholesterol, or patient-reported outcomes. It may be a useful alternative to usual care for diabetes management [8]. A study by Mossad et al. (2024) in Egypt demonstrated the success of a six-month tele-geriatrics program for dementia patients and caregivers. Among 70 participants, the intervention reduced delirium, behavioral symptoms, clinic and ER visits, and caregiver stress, highlighting teleconsultation effectiveness in geriatric care [9].

Telediagnosis enables healthcare providers to remotely assess medical conditions using high-resolution images and data from wearable or monitoring devices. It may occur synchronously or asynchronously [10]. A recent meta-analysis of 44 studies (2023) found a pooled diagnostic agreement of about 69% (κ ≈ 0.67) between teledermatology and in-person assessments, with higher concordance when evaluations were performed by dermatologists and when digital imaging and training were used. These results support teledermatology as a useful diagnostic tool, particularly in resource-limited settings. However, the evidence is constrained by heterogeneity across studies, modest agreement with histopathology (55.7%), limited lesion-specific data, and variable methodological quality, which together limit the generalizability of findings [11].

Telemonitoring tracks patients’ health via connected devices (e.g., smartwatches, glucometers), transmitting data to healthcare providers for timely interventions. Particularly effective in chronic disease management, telemonitoring helps reduce hospital readmissions [12]. A systematic review of 91 studies found that remote patient monitoring reduced hospital admissions and emergency visits, particularly for cardiovascular and respiratory diseases [13]. Kamel et al. (2021) studied telemonitoring post-PCI in 200 cardiac patients. Though no significant difference in major cardiac events was observed, adherence to statins, ACE inhibitors, and lifestyle changes improved in the telemedicine group, with high patient satisfaction [14].

Tele-treatment includes remote delivery of therapy, such as physical rehabilitation, mental health counseling, and even telesurgery. It enables patients to access expert guidance or surgical interventions without traveling [15]. In a meta-analysis by Gurcay et al. (2023), telehealth interventions significantly improved medication adherence among patients with type 2 diabetes mellitus, supporting its integration into chronic disease care [16]. A systematic review of 35 randomized controlled trials evaluating CBT-informed digital interventions for adults with depression and anxiety found that internet- and mobile-based programs are feasible and can be effective, particularly when compared with waitlist controls. Benefits were greater when interventions included clinical support or were combined with other treatments. While many trials were small and short-term, evidence supports their use as low-intensity tools for subclinical symptoms, a first step in stepped-care models, and a cost-effective, accessible option for preventive programs [17].

Telerehabilitation allows patients, especially older adults or those with chronic diseases, to undergo remote physiotherapy, nutritional counseling, and occupational therapy [18]. A systematic review of 15 RCTs on older adults with knee osteoarthritis found telerehabilitation superior to usual care in improving pain, physical function, and mobility, though effects on mental health and quality of life were not significant [19]. Rennie et al. reported that telerehabilitation generated average cost savings of £38.57 per patient, primarily due to reduced travel requirements [20].

Tele-education offers older adults tailored health information and self-care education on topics like nutrition, medication adherence, and mental health. It promotes autonomy and informed decision-making, ultimately improving their health and quality of life. Tele-education messages can be delivered through a variety of media, including text messages, mobile applications, video calls, social media platforms, and television or radio programs. For older adults with limited literacy skills, voice-based and visual media are particularly effective. Combining these methods with culturally appropriate illustrations and caregiver involvement can further enhance comprehension and ensure equitable access to health education [21, 22].

## Mobile health (mHealth) in older adult care

mHealth refers to the use of mobile devices such as smartphones, tablets, and wireless sensors in delivering healthcare services and supporting public health. First introduced in 2003, mHealth is now a critical component in older adult care, offering benefits like remote monitoring, medication management, and enhanced emergency response [23].

mHealth apps enable continuous monitoring of vital signs such as heart rate, glucose levels, and physical activity. For example, Kitsiou et al. (2017) reviewed 15 systematic reviews on diabetes-related mHealth interventions and found that these tools improved glycemic control, with HbA1c levels decreased by up to 0.8% in type 2 diabetes patients. Additionally, predictive analytics are being integrated into apps [24]. Larburu et al. developed an AI-based app using patient data and a Naive Bayes model to forecast heart failure risks, aiding early intervention [25].

Older adults often struggle with medication adherence due to complex regimens. mHealth apps can offer reminders, track doses, and notify about refills. A review of 11 studies found that seven demonstrated improved adherence through app use, with high user satisfaction and ease of use reported, indicating mHealth’s potential for enhancing medication compliance [26].

Emergency alert features, including fall detection and GPS tracking, allow mHealth apps to notify caregivers and emergency services promptly. Wang et al. (2020) reported that such systems significantly reduced emergency response times for older adults experiencing falls, improving safety and outcomes [27].

mHealth apps support self-care by enabling patients to communicate with providers, access health education, and receive feedback. A systematic review and meta-analysis of 41 randomized controlled trials evaluated mobile health applications for diabetes management. App-based interventions significantly improved glycemic control, reducing HbA1c by 0.49% (95% CI: − 0.65 to − 0.32%) and enhancing self-management behaviors compared to standard care. While some heterogeneity and moderate risk of bias were noted, the findings support the use of mobile apps as effective tools in diabetes care, with future research needed to standardize app features and improve study quality [28].

Beyond physical care, mHealth applications also promote social and emotional well-being. Apps that enable video calls and virtual communities help reduce social isolation. A gamified app used by community-dwelling seniors with frailty improved social connectedness and decreased loneliness, highlighting the value of emotional support features [29].

mHealth platforms help caregivers coordinate care through features like shared calendars, medication trackers, and health record storage. *CareZone*. A systematic review of 28 studies showed that mHealth tools significantly reduce caregiver burden and enhance well-being [30]. However, Castillo et al. (2024) noted that while some dementia caregiver apps offer value, many still fall short in addressing complex caregiving needs [31].

## Integration of artificial intelligence in healthcare delivery

Artificial Intelligence (AI) is revolutionizing healthcare by analyzing large, diverse datasets—such as electronic health records, genetic profiles, and real-time patient data—to enable predictive, personalized, and proactive care. AI includes techniques like machine learning (ML), deep learning (DL), and natural language processing (NLP). Large Language Models (LLMs), such as ChatGPT, are AI systems capable of understanding and generating human-like text [32].

In geriatric care, AI supports early identification of risks such as falls, enhances diagnosis accuracy, predicts disease progression, and assists in treatment planning. Operationally, AI helps forecast patient admissions and optimize hospital resources, while its integration with telehealth systems allows for real-time alerts, reducing avoidable hospitalizations [33].

AI enhances medical diagnostics, especially through image analysis, revolutionizing diagnostic imaging by improving accuracy, efficiency, and personalized healthcare delivery [34]. In Alzheimer’s disease research, Jo et al. developed a hybrid model combining DL and ML to analyze MRI scans, achieving a diagnostic accuracy of 96%. These tools enable earlier and more accurate detection, which is vital in age-related diseases [35].

AI enables precision medicine by analyzing patients’ genetic data, lifestyle, and history to recommend individualized treatment plans. In cancer care, AI is being used to predict treatment responses based on tumor characteristics. This approach supports tailored interventions, drug dosage optimization, and improved clinical outcomes [36].

AI-driven chatbots and voice assistants serve as virtual health aids, scheduling appointments, answering health questions, or offering mental health support. These tools are especially useful in chronic disease management and mental wellness, providing scalable, around-the-clock interaction and support [37].

AI-powered Clinical Decision Support (CDS) systems leverage ML and NLP to support clinicians by interpreting complex data and suggesting evidence-based decisions. A systematic review of 20 studies highlighted benefits such as better patient evaluation and time efficiency but noted barriers like workflow misalignment, usability issues, and clinician trust. The findings underscore the need for user-centered design in CDS tools [38].

AI accelerates drug discovery by predicting drug-target interactions, identifying new molecules, and optimizing trial design. Research resources such as AlphaFold and AlphaMissense have expanded the possibilities for biomedical discovery [39, 40]. AlphaFold provides an open database of more than 200 million predicted protein structures, greatly accelerating structural biology and supporting drug discovery research, though it is not yet a validated clinical tool [39]. AlphaMissense is a novel AI model that predicts the pathogenicity of all possible human missense variants. Recently published findings indicate that AlphaMissense effectively integrates structural information from AlphaFold to enhance its predictions. This tool is particularly valuable for guiding future genetic studies, although it is not currently deployed in clinical diagnostics [40].

AI can detect individuals at high risk for diseases such as stroke or coronary artery disease. A review analyzing over 100 studies and 3.4 million participants found that ML algorithms achieved strong predictive performance, boosting algorithms reached an Area Under the Curve (AUC) of 0.88 for coronary artery disease, while support vector machines achieved 0.92 for stroke prediction [41].

AI chatbots are emerging as tools for health education, especially in geriatrics. They provide tailored health information, encourage self-care, and answer complex health questions in user-friendly formats [42]. Studies show that chatbots improve patient engagement and knowledge retention. Tools like ChatGPT-4 demonstrate strong accuracy, though continued improvements are needed to ensure consistency across medical domains [43]. Systematic reviews have found that AI-driven tools can enhance caregivers’ abilities by analyzing patient data, improving the quality and consistency of home-based care. These tools do not replace caregivers but serve to augment their capabilities and reduce physical and emotional strain, offering pathways to more sustainable care delivery [44].

## Innovative assistive technologies for older adults

Advances in portable health technologies are transforming healthcare for older adults by enabling real-time monitoring, early detection of diseases, and fostering independent living. Devices such as wearables, smart textiles, and patch-based monitors collect physiological data to support proactive health management [45].

### Wearable devices for older adults

Smart rings like the Oura Ring monitor vital signs such as heart rate, temperature, and sleep quality. A trial of a ring-based, cuffless blood pressure monitor showed strong accuracy compared to traditional methods (mean SBP difference: 0.16 mmHg; DBP: −0.07 mmHg), making it a viable tool for home and clinical use [46].

Smartwatches track movement, heart rate, and sleep, automatically syncing data to healthcare systems. They can promote healthy behavior through nudges and predict risks based on long-term monitoring. A 2022 study showed smartwatch-based fall detection achieved 77% sensitivity and 99% specificity [47]. Apple’s MM4PD app detects Parkinson’s symptoms like tremors and dyskinesia, helping personalize treatment [48].

Early attempts at non-invasive glucose monitoring included the *GlucoWatch*, which received FDA approval in 2001 but was discontinued in the mid-2000s due to limitations such as skin irritation and performance issues. Current continuous glucose monitoring (CGM) systems, such as Dexcom, FreeStyle Libre, and Eversense, represent the state of the art. These devices are minimally invasive, relying on subcutaneous sensors to provide real-time glucose monitoring and alerts, and have demonstrated substantial benefits in glycemic control and patient self-management [49].

Smart glasses and contact lenses enhance communication and health tracking. Kum et al. created a smart contact lens that measures tear glucose and delivers drugs to treat diabetic retinopathy. In rabbit models, glucose readings matched traditional tests, showing the potential for real-time disease management [50].

Patch sensors like the AT-Patch and Zio^®^ Patch provide continuous heart monitoring. The AT-Patch identified atrial fibrillation in high-risk users with a strong agreement score (κ = 0.840) [51]. The Zio Patch detected 57% more events than standard Holter monitors. Other patches monitor sleep apnea by tracking heart rate and chest movement, offering non-invasive diagnostics with high accuracy [52].

Textiles with embedded sensors track ECG, posture, and falls. Smart shirts and compression garments offer real-time feedback, supporting remote monitoring and independence [53]. The Cyrcadia Breast Monitor (CBM) uses temperature-sensing patches to detect circadian pattern changes linked to breast cancer. In clinical studies with 201 participants, it showed 83.6% sensitivity and 71.5% specificity [54]. Smart insoles monitor gait patterns in Parkinson’s disease and can detect abnormal movements before a formal diagnosis. Combined with machine learning, these systems help providers manage care more effectively [55].

### Assistive robotics and socially assistive robotics (SAR)

Assistive robotics and socially assistive robotics (SAR) are transforming geriatric care by providing support that enhances independence, safety, and emotional well-being among older adults. These technologies serve various functions, including physical assistance, social engagement, and cognitive support, helping older adults live more autonomously while alleviating the burden on caregivers [56].

Pepper, a humanoid robot developed by SoftBank Robotics, has been commercially available and tested in limited pilot implementations rather than being widely adopted. A qualitative case study explored Pepper’s use in rehabilitation activities for older adults with schizophrenia or dementia in Japan. It found that the robot could encourage simple exercises and brief conversations, promoting positive attitudes and calmness. However, it concluded that Pepper’s technology still requires major improvements to better support communication, engagement, and therapeutic benefits in long-term care [57]. Another qualitative pilot study explored the experiences of residents and healthcare professionals in a Norwegian long-term care facility interacting with the Pepper social robot. Both residents and staff enjoyed Pepper’s presence and found it added variety and engagement to daily routines. However, they also expressed concerns about practical challenges, ethical issues, and technological limitations, noting that while social robots show potential to enrich care activities, their development and integration in healthcare remain at an early, experimental stage [58].

Therapeutic robots such as Paro, a baby-seal robot equipped with tactile, auditory, and light sensors, have demonstrated reductions in stress, agitation, and depressive symptoms among older adults, especially those with dementia [59]. A recent systematic review and meta-analysis (2023) examined the effectiveness of the Paro therapeutic robot for older adults with dementia across 12 trials involving 1,461 participants. Results showed that Paro produced a moderate reduction in medication use and small improvements in anxiety, agitation, and depression, but had minimal impact on sleep duration. Narrative findings also indicated reduced apathy and greater sociability. However, the overall evidence quality was low due to small sample sizes, varied study designs, and methodological limitations. The authors concluded that Paro shows promise as a non-pharmacological intervention for managing behavioral and psychological symptoms of dementia, but emphasized the need for larger, more rigorous studies to confirm its effectiveness and identify optimal implementation approaches [59].

Similarly, Zora, a humanoid robot used in nursing homes to facilitate physical therapy sessions and mobility exercises [60], was examined in a Finnish case study exploring its long-term integration in elderly care using domestication theory. Initially, Zora enhanced engagement and enthusiasm among staff and residents; however, long-term adoption was limited by technical challenges, insufficient training, and a lack of organizational commitment. The study highlighted that sustainable implementation of care robots requires systematic integration and strong institutional support beyond pilot initiatives [61].

Robotic exoskeletons have shown promise in supporting individuals with reduced mobility or neurodegenerative diseases [62]. A randomized controlled trial investigated the Kickstart^®^ Walk Assist system in 46 stroke patients. Participants who used the device in addition to standard therapy showed greater improvements in lower limb motor function, muscle activity, and fatigue reduction over 8 weeks compared to those receiving conventional training alone. While gains in walking speed and gait parameters were modest, no adverse effects occurred. The findings suggest that Kickstart^®^ is a safe, practical, and beneficial supplement to rehabilitation, helping enhance leg strength and walking ability in stroke recovery [63].

In the cognitive domain, social robots use facial and voice recognition to personalize interactions and deliver cognitive games and reminders. A randomized controlled trial in Korea found that a 6-week social robot PIO program significantly improved cognitive function among older adults with mild to moderate dementia in daycare centers (*p* <.001). However, its effect on depression was not statistically significant, suggesting the need for further research [64]. On the other hand, A meta-analysis of 10 studies (2015–2021) evaluated robot interventions for older adults with cognitive impairment. showed that robots significantly reduced anxiety and agitation but had no significant effect on cognitive function, neuropsychiatric symptoms, or quality of life. Pet-type robots and individualized interventions were most effective for improving psychological outcomes, highlighting the need for further research on cognitive effects [65].

During the COVID-19 pandemic, telepresence robots such as GiraffPlus supported virtual communication and monitoring between older adults and caregivers [66]. A Canadian study explored the experiences of 20 care partners using telepresence robots in long-term care homes during the COVID-19 pandemic. Findings showed that robot use reduced caregiver burden, improved relationships with staff, and enhanced autonomy and communication with residents. Overall, telepresence robots were viewed as valuable tools for supporting ongoing caregiving, though broader studies are needed to assess long-term sustainability [67].

### Virtual reality (VR)

Virtual reality (VR) technologies are emerging as powerful tools in elder care, offering innovative ways to address cognitive decline, social isolation, physical inactivity, and psychological distress. VR provides immersive environments that support various forms of therapeutic engagement. One key application is social interaction through virtual group activities, such as simulated cycling or group tours, which can enhance mood, cognitive stimulation, and a sense of community [68].

In the realm of cognitive training, VR-based interventions have shown promising results in improving memory and executive function. For instance, reminiscence therapy conducted through VR allows older adults to revisit familiar places or experiences, fostering emotional well-being and cognitive engagement [69]. A recent 2022 study demonstrated that VR-based cognitive-motor rehabilitation significantly outperformed traditional training programs in enhancing cognitive outcomes in older adults [70].

VR also promotes physical health by offering engaging, gamified exercise programs (exergames), which reduce sedentary behavior and encourage movement [71]. Moreover, VR has been successfully used to manage anxiety and pain. For example, combining nature-based VR with heart rate variability biofeedback (HRVBF) has helped alleviate preoperative anxiety and postoperative pain among older adults undergoing knee arthroplasty [72].

User acceptance plays a critical role in the successful implementation of VR in elder care. Research shows that older adults tend to favor realistic, life-like environments over cartoonish or abstract simulations. While advanced systems like omnidirectional treadmills can enhance engagement, they often require initial training and support. Co-design approaches that involve older adults in the development process are essential to ensure usability, safety, and satisfaction with VR tools [73].

### Smart homes

Smart home technologies are reshaping how older adults age in place by integrating artificial intelligence (AI), Internet of Things (IoT) devices, and sensor-based monitoring systems. These technologies aim to create responsive, adaptive environments that enhance safety, support health monitoring, and promote social engagement. Smart homes continuously collect and analyze data on vital signs, movement patterns, and environmental conditions to detect anomalies and provide timely interventions. For instance, automated alerts can be sent to caregivers in the event of a fall or abnormal heart rate, reducing the need for emergency hospital visits [74].

Safety is a central focus of smart home design. Features such as adaptive lighting, voice-controlled appliances, fall-prevention flooring, and automated doors and furniture help maintain mobility and reduce accident risks. Smart homes also support cognitive and emotional well-being through devices like voice assistants and smart speakers, which deliver personalized reminders, memory exercises, and interactive games tailored to individual needs [75].

Beyond individual support, smart home technologies enhance social inclusion by enabling virtual communication with family members, caregivers, and peers [76]. AI-powered systems can monitor mood, detect signs of depression, and trigger appropriate social or medical interventions. Some smart platforms also integrate socially assistive robots and virtual companions, providing both emotional support and structured daily routines [77].

These innovations represent a significant shift in elder care, blending technology and personalized support to improve quality of life. However, ensuring accessibility, affordability, and user-friendliness remains essential to maximize their impact, particularly in low-resource settings.

## Challenges of telemedicine and AI in healthcare

Despite their transformative potential, telemedicine and AI face several critical implementation challenges. One of the most significant concerns is privacy and data security. Patients are increasingly wary of how sensitive health data is collected, stored, and transmitted, particularly during virtual consultations. Studies show that many health apps, especially those targeting dementia prevention, lack transparency in data handling, expert involvement, and evidence-based content [78]. These gaps compromise user trust and violate essential ethical standards. AI systems, which require large volumes of patient data to function effectively, raise additional concerns regarding cybersecurity and informed consent. Balancing innovation with robust privacy protections and ethical governance is therefore essential for sustainable implementation [78].

Another major challenge is equity and access. Although global internet connectivity has improved, a substantial digital divide persists, disproportionately affecting older adults, rural populations, and low-income communities. According to the International Telecommunication Union (ITU), about 68% of the world’s population was online in 2024, leaving approximately 2.6 billion people still offline. Internet penetration remains highly unequal, 87% in Europe versus only 33% in Africa, and large gaps persist between urban (82%) and rural (44%) populations globally [79].

Moreover, internet affordability remains a major challenge. In several low- and middle-income countries, the cost of 1 GB of mobile data exceeds 2% of monthly income, and in low-income economies, fixed broadband subscriptions can consume nearly one-third of average earnings. In least-developed countries, mobile broadband costs represent about 4.2% of income compared to only 0.3% in Europe, reflecting stark global inequities that may further hinder access to digital health and telemedicine services [80].

Older adults, particularly those with low education and health literacy, also experience technophobia and usability challenges. Addressing these inequalities require targeted digital literacy programs, affordable connectivity initiatives, and culturally sensitive strategies to ensure inclusive access to telehealth technologies [81].

 Legal and regulatory considerations also impede the broad adoption of telehealth and AI. Cross-jurisdictional licensing, inconsistent reimbursement policies, and unclear liability protocols create legal ambiguities for providers and patients. Establishing comprehensive standards that address clinical protocols, data privacy, and malpractice liability is essential for ensuring care quality and safety in virtual settings [82].

Equally important are the trust issues associated with AI systems. Many AI tools function as “black boxes,” where the decision-making process is opaque to users and clinicians. Lack of explanation and embedded biases, arising from flawed training data or model design, further diminish trust in AI systems. Overcoming this issue requires transparency in algorithm design, routine audits, and communication strategies that demystify AI-generated recommendations. Trust can be built through user-centered design and active engagement with both clinicians and patients [83].

AI development is also constrained by data scarcity. High-quality, annotated medical datasets are difficult to obtain due to fragmentation across health systems, labeling bottlenecks, privacy restrictions, and high collection costs. Rare diseases present additional challenges, as small sample sizes undermine model accuracy. Addressing these limitations demands collaborative data-sharing frameworks, standardized formats, and systems-level thinking to ensure the development of robust, ethical, and clinically relevant AI tools [84].

While mHealth technologies provide opportunities for older adults, usability barriers remain significant. Cognitive decline can hinder app navigation, motor impairments (e.g., arthritis) affect device handling, and sensory deficits necessitate adaptations such as larger fonts, high-contrast displays, audio cues, and voice controls. Motivation and confidence in using technology are often low, but strategies such as gamification, intuitive interfaces, and privacy education can enhance adoption. Involving older adults in the design process is essential to ensure tools are both accessible and relevant [85].

Similarly, SARs face acceptance challenges. Privacy concerns, limited emotional intelligence, and user discomfort with robotic appearance or behavior restrict their integration. Studies show that older adults prefer empathetic, small, animal-like robots operating in familiar environments. These insights highlight the importance of user-centered, culturally sensitive design to improve acceptance and maximize the benefits of SAR technologies [86].

## Key considerations for designing technology for older adults

Designing effective and inclusive technologies for older adults requires a multidisciplinary and empathetic approach. Engineers, psychologists, and computer scientists must consider the unique cognitive, physical, and emotional needs of older users. Key design principles include usability, simplicity, predictability, and efficiency. Technologies should feature intuitive interfaces, ergonomic designs, and sensory feedback mechanisms (e.g., voice prompts with appropriate tone and clarity). Customizability is also essential, allowing older users to adapt systems to their preferences and limitations. Research highlights that older adults prefer friendly, familiar designs such as robots resembling pets or humans, which encourage acceptance and interaction [87].

User-centered design studies have shown that older adults are willing to engage in the co-creation of technology, provided their concerns around control, data privacy, and usability are addressed. Focus groups with retirement community residents identified barriers such as low-tech literacy and physical challenges but also revealed enthusiasm for learning and co-design. Bridging the gap between older users and technologists requires raising technology literacy while promoting aging sensitivity among designers [88].

This review is a narrative (non-systematic) synthesis intended to provide an overview of key innovations and emerging trends in geriatric care rather than a comprehensive systematic evidence synthesis. The examples and studies cited were selected purposively to highlight representative approaches, technologies, and models of care that reflect recent advancements and relevance to the field. As such, potential selection bias cannot be excluded, and the presented evidence should be considered illustrative rather than exhaustive. Future systematic reviews are recommended to validate and expand upon these insights.

## Conclusion

The rapid aging of the global population presents significant challenges for healthcare systems, particularly with the growing prevalence of chronic and degenerative diseases among older adults. These conditions require coordinated, personalized, and long-term approaches beyond traditional care models. Technological innovations such as telemedicine, mHealth, robotics, and AI offer promising tools to meet these needs. Telemedicine improves access to healthcare, especially in remote areas, while mHealth tools support self-management and early detection. Robotics enhance daily functioning, cognitive stimulation, and emotional well-being, and AI facilitates data-driven, personalized care planning and decision-making.

These advancements not only support older adults but also reduce caregiver burden by automating routine tasks and improving care coordination. However, barriers such as technical limitations, data privacy concerns, and unequal access remain critical. Such barriers underscore that digital innovations may unintentionally widen health inequities unless equity is prioritized in implementation.

Good-practice policy suggestions include expanding broadband infrastructure and improving affordability in low- and middle-income regions to reduce connectivity gaps; establishing national telehealth frameworks to standardize practice and ensure quality; and offering targeted digital literacy and training programs for older adults and caregivers. Subsidies for low-income older adults could also improve access to assistive technologies, while clear national standards and data protection regulations are essential to safeguard ethical AI and robotics use in geriatric care.

Future research should focus on large-scale comparative studies evaluating the effectiveness, safety, and cost-efficiency of these technologies relative to traditional care models. Further evidence is also needed on how socioeconomic, cultural, and gender factors influence technology adoption and sustained use. Priority should be given to co-design approaches that actively involve older adults and caregivers, ensuring interventions are user-centered, culturally appropriate, and equitable.

## Data Availability

No datasets were generated or analysed during the current study.
